# Misperception influence on zero-determinant strategies in iterated Prisoner’s Dilemma

**DOI:** 10.1038/s41598-022-08750-8

**Published:** 2022-03-25

**Authors:** Zhaoyang Cheng, Guanpu Chen, Yiguang Hong

**Affiliations:** 1 Key Laboratory of Systems and Control, Academy of Mathematics and Systems Science, Beijing, 100190 China; 2 School of Mathematical Sciences, University of Chinese Academy of Sciences, Beijing, 100190 China; 3 JD Explore Academy, Beijing, 100176 China; 4 Present Address: Department of Control Science and Engineering, Tongji University, Shanghai, 201804 China

**Keywords:** Stochastic modelling, Applied mathematics, Information technology, Environmental economics

## Abstract

Zero-determinant (ZD) strategies have attracted wide attention in Iterated Prisoner’s Dilemma (IPD) games, since the player equipped with ZD strategies can unilaterally enforce the two players’ expected utilities subjected to a linear relation. On the other hand, uncertainties, which may be caused by misperception, occur in IPD inevitably in practical circumstances. To better understand the situation, we consider the influence of misperception on ZD strategies in IPD, where the two players, player *X* and player *Y*, have different cognitions, but player *X* detects the misperception and it is believed to make ZD strategies by player *Y*. We provide a necessary and sufficient condition for the ZD strategies in IPD with misperception, where there is also a linear relationship between players’ utilities in player *X*’s cognition. Then we explore bounds of players’ expected utility deviation from a linear relationship in player *X*’s cognition with also improving its own utility.

Iterated Prisoner’s Dilemma (IPD) games have long been studied for understanding the evolution of cooperation and competition between players^1–3^. It is generated by a one-shot Prisoner’s Dilemma (PD) game between player *X* and player *Y*, where both of them choose to cooperate (c) or defect (d). Players’ utility matrix is shown in Table 1, where parameters [*T*, *R*, *P*, *S*] of the PD game are constrained by $T>R>P>S$ and $2R>T+S$^4–6^. Thus, mutual defection is the only Nash equilibrium, but mutual cooperation is the globally best outcome. In IPD games, the analysis of players’ utilities is quite complicated since players may promote cooperation through past actions. Fortunately, Press and Dyson^7^ proposed zero-determinant (ZD) strategies, where the player equipped with ZD strategies can unilaterally enforce the two players’ expected utilities subjected to a linear relation. Afterward, various ZD strategies were widely studied in public goods game (PGG), human-computer interaction (HCI), and moving target defense (MTD) problems^8–11^. For example, the equalizer strategy^7,12^ is a special ZD strategy that can unilaterally set the opponent’s utility. Besides, the player who adopts extortion strategies^7,13^ can make that its utility is not lower than the opponent’s utility. Conversely, the generous strategy^14,15^ is another special ZD strategy that ensures that the utility of the player with generous strategies is not higher than the opponent’s utility, but it is dominant in the game.

**Table 1:** Utility matrix in PD games.

		*Y*	
		c	d
*X*	c	(*R*, *R*)	(*S*, *T*)
	d	(*T*, *S*)	(*P*, *P*)

Actually, uncertainty is always unavoidable in human interactions^16^, and there have been many models to describe uncertain circumstances in game theory, such as robust games, stochastic games, and hypergames^17–19^. Misperception is one of the most common uncertain phenomena. For example, in the Internet of Things, limited attention is a type of misperception, leading to bounded rationality and increasing cyber risks of the community^20^, and in cyber security problems, hackers may have a confused cognition of the system’s TCP/IP stack, which is known to the network administrator^21^. Moreover, players’ strategies may be influenced by uncertainty, which results in obvious deviation from opponents’ cognitions and attendant suspicion, such as the extenuating circumstances which consider intentions and outcomes in the legal system^22,23^, while players may misunderstand their opponents’ strategies, such as some companies relying on private monitoring instead of their opponents’ real actions^24,25^.

In fact, the condition for players to trust their cognition is crucial in games with misperception^26,27^. Particularly, misperception may spoil players’ cognition if others’ strategies are not consistent with their own anticipation, and moreover, it may even ruin the balance or even lead to collapse of the model^28^. For instance, in psychological experiments, participants’ doubts may affect the sponsor’s control^29^. Actually, due to the historical information or knowledge from others, a player may know that its opponent takes some given strategies, and moreover, the awareness of the opponent’s ZD strategies has been widely considered in many IPD games^7,9^. In the case when players prefer ZD strategies in IPD with misperception, a player may doubt its cognition if its opponent does not choose ZD strategies as it expects. Nevertheless, most existent works on ZD strategies in IPD with uncertainties, such as ZD strategies with observation errors^24,25^ or implementation errors^22,30^, have paid less attention to strategies that maintain players’ cognition.

Therefore, the motivation of this paper is to analyze how misperception affects a player’s ZD strategy without causing its opponent’s suspicion. Specifically, we consider the case when player *X* knows the misperception about the game, and player *Y* believes that player *X* prefers to make ZD strategies according to the original model without misperception. Then player *X* tends to choose strategies consistent with its opponent’s anticipation, and meanwhile improve its own expected utility.

To this end, we find some conditions where player *X* is able to achieve at least a linear relationship between players’ expected utilities without causing the opponent’s awareness of misperception. Additionally, misperception can bring a bounded deviation from the linear relationship between players’ expected utilities in player *X*’s cognition, which can be applied to player *X*’s strategy implementation. Further, player *X* can utilize the misperception and take some benefits, such as improving the supremum or the infimum of its expected utility.

## Results

### Models

Consider an IPD game with misperception such as implementation errors and observation errors^22,23,31^. Due to the misperception, the parameter in the real game changes from $\omega _1=[T_1,R_1,P_1,S_1]$ to $\omega _2=[T_2,R_2,P_2,S_2]$, and only player *X* notices the change. Thus, player *Y*’s cognition of the parameter is $\omega _1$, while player *X*’s cognition of the parameter is $\omega _2$. In each round, player *X* chooses a strategy from its strategy set $\Omega _X=\{{\mathbf {p}}=[p_{cc},p_{cd},p_{dc},p_{dd}]^T|p_{xy} \in [0,1],xy\in \{cc,cd,dc,dd\}\}$, *e.g.*, $p_{xy}$ is player *X*’s probability for cooperating with given previous outcome $xy\in \{cc,cd,dc,dd\}$. Similar to $\Omega _X$, player *Y*’s strategy set is $\Omega _Y=\{{\mathbf {q}}=[q_{cc},q_{dc},q_{cd},q_{dd}]^T|q_{xy} \in [0,1],xy\in \{cc,dc,cd,dd\}\}$. According to Press and Dyson^7^, this game can be characterized by a Markov chain with a state transition matrix $M=[M_{jk}]_{4\times 4}$ (see “Notations” for details). Denote ${\mathbf {v}}=[v_{cc},v_{cd},v_{dc},v_{dd}]^T$ as a probability vector such that ${\mathbf {v}}^T M={\mathbf {v}}^T$ and $v_{cc}+v_{cd}+v_{dc}+v_{dd}=1$. Let ${\mathbf {S}}^{\omega _i}_{X}=[R_i,S_i,T_i,P_i]^T$, and ${\mathbf {S}}^{\omega _i}_{Y}=[R_i,T_i,S_i,P_i]^T,$ $i\in \{1,2\}$. The expected utility functions of players are as follows:

$$\begin{aligned} \begin{aligned} u_X^{\omega _i}({\mathbf {p}},{\mathbf {q}})={\mathbf {v}} \cdot {\mathbf {S}}^{\omega _i}_{X}, \ u_Y^{\omega _i}({\mathbf {p}},{\mathbf {q}})={\mathbf {v}} \cdot {\mathbf {S}}^{\omega _i}_{Y},i\in \{1,2\}. \end{aligned} \end{aligned}$$

Denote $G_1 = \{{\mathbf {P}}, {\varvec{\Omega }}, {\mathbf {u}}, \omega _1\}$, and $G_2=\{{\mathbf {P}},{\varvec{\Omega }},{\mathbf {u}},\omega _2\}$, where ${\mathbf {P}}=\{X,Y\}$, ${\varvec{\Omega }}=\Omega _X\times \Omega _Y$, and ${\mathbf {u}}=\{u_X^{\omega _i},u_Y^{\omega _i}\}, i\in \{1,2\}$. Thus, the actual utilities of players are obtained through $G_2$, and in the view of player *Y*, they are playing game $G_1$. In the view of player *X*, they are playing game $G_2$ but player *X* knows that player *Y*’s cognition is $G_1$. $G_1$ and $G_2$ are shown in Table 2.

**Table 2:** Utility matrices in IPD games with misperception.

(a) $G_1$				(b) $G_2$			
		*Y*				*Y*	
		c	d			c	d
*X*	c	$(R_1,R_1)$	$(S_1,T_1)$	*X*	c	$(R_2,R_2)$	$(S_2,T_2)$
	d	$(T_1,S_1)$	$(P_1,P_1)$		d	$(T_2,S_2)$	$(P_2,P_2)$

Let ${\mathbf {p}}_0=[1,1,0,0]^T$. For $i\in \{1,2\}$, ${\mathbf {p}}=\alpha {\mathbf {S}}^{\omega _i}_{X} +\beta {\mathbf {S}}^{\omega _i}_Y +\gamma {\mathbf {1}}+{\mathbf {p}}_0$, where $\alpha ,\beta ,\gamma \in {\mathbb {R}}$, is called a *ZD strategy*^7^ of player *X* in $G_i$ since the strategy makes the two players’ expected utilities subjected to a linear relation:

$$\begin{aligned} \alpha u_X^{\omega _i}({\mathbf {p}},{\mathbf {q}})+\beta u_Y^{\omega _i}({\mathbf {p}},{\mathbf {q}})+\gamma =0, \end{aligned}$$

for any player *Y*’s strategy ${\mathbf {q}}$. All available ZD strategies for player *X* in *G* can be expressed as $\Xi (\omega _i)=\{{\mathbf {p}}\in \Omega _X|{\mathbf {p}}=\alpha {\mathbf {S}}^{\omega _i}_{X} +\beta {\mathbf {S}}^{\omega _i}_Y +\gamma {\mathbf {1}}+{\mathbf {p}}_0,\alpha ,\beta ,\gamma \in {\mathbb {R}} \}.$ Also, the three special ZD strategies are denoted as:

1. equalizer strategy^7,12^: $${\mathbf {p}}=\beta {\mathbf {S}}^{\omega _i}_{Y}+\gamma {\mathbf {1}}+{\mathbf {p}}_0$$;
2. extortion strategy^7,13^: $${\mathbf {p}}=\phi [({\mathbf {S}}^{\omega _i}_X-P_i{\mathbf {1}})-\chi ({\mathbf {S}}^{\omega _i}_Y-P_i{\mathbf {1}})]+{\mathbf {p}}_0,\chi \geqslant 1$$;
3. generous strategy^14,15^: $${\mathbf {p}}=\phi [({\mathbf {S}}^{\omega _i}_X-R_i{\mathbf {1}})-\chi ({\mathbf {S}}^{\omega _i}_Y-R_i{\mathbf {1}})] +{\mathbf {p}}_0,\chi \geqslant 1$$.

Based on the past experience, player *Y* knows that player *X* prefers ZD strategies, which has been widely considered in many IPD games^7,9^. To avoid that player *Y* notices the change, which may result in potential decrease of player *X*’s utility^21^ or collapse of the model^28^, player *X* keeps choosing ZD strategies according to $G_1$, such that the strategy sequence matches player *Y*’s anticipation. To sum up, in our formulation,

- the real game is $G_2$;
- player *Y* thinks that they are playing game $G_1$, and player *X* thinks that they are playing game $G_2$;
- player *X* knows that player *Y*’s cognition is $G_1$;
- player *Y* believes that player *X* chooses ZD strategies;
- player *X* tends to choose a ZD strategy according to $G_1$ to avoid player *Y*’s suspicion of misperception.

In fact, player *X* can benefit from the misperception through the ZD strategy. For example, player *X* can adopt a generous strategy in $G_1$ to not only promote player *Y*’s cooperation behavior, but also make player *X*’s utility higher than that of player *Y*, if the generous strategy is an extortion strategy in $G_2$. A beneficial strategy for player *X* is able to maintain a linear relationship between players’ utilities or improve the supremum or the infimum of its utility in its own cognition. In the following, we aim to analyze player *X*’s implementation of a ZD strategy in IPD with misperception, and proofs are given in the Supplementary Information.

### Invariance of ZD strategy

Player *X*’s ZD strategies may be kept in IPD games with misperception from implementation errors or observation errors. In particular, player *X* keeps choosing a ZD strategy ${\mathbf {p}}$ in $G_1$ to avoid player *Y*’s suspicion about possible misperception. In the view of player *X*, it can also enforce players’ expected utilities subjected to a linear relationship if ${\mathbf {p}}$ is also a ZD strategy in $G_2$. The following theorem provides a necessary and sufficient condition for the invariance of the linear relationship between players’ utilities.

#### Theorem 1

Any ZD strategy ${\mathbf {p}}$ of player *X* in $G_1$ is also a ZD strategy in $G_2$ if and only if

1 $$\begin{aligned} \frac{R_1-P_1}{2R_1-S_1-T_1}=\frac{R_2-P_2}{2R_2-S_2-T_2}. \end{aligned}$$

**Figure 1:**
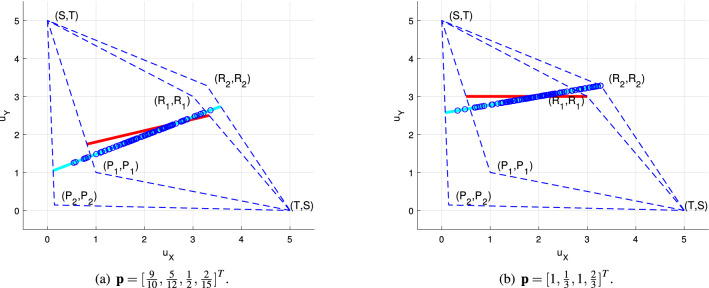
Player *X* can also enforce a linear relationship between players’ utilities in its own cognition. Let $\omega _1=[T,R_1,P_1,S]=[5,3,1,0]$ and $\omega _2=[T,R_2,P_2,S]=[5,\frac{23}{7},\frac{1}{7},0]$, which satisfy (1). Consider that player *X* chooses two different ZD strategies in (**a**) and (**b**), respectively, and the red lines describe the relationships between players’ utilities in $G_1$. We randomly generate 100 player *Y*’s strategies, and blue circles are $(u^{\omega _2}_X,u^{\omega _2}_Y)$, correspondingly. Notice that blue circles are indeed on a cyan line in both (**a**) and (**b**).

If (1) holds, player *X* can ignore the misperception and choose an arbitrary ZD strategy based on its opponent’s anticipation since it also leads to a linear relationship between players’ utilities, as shown in Fig. 1; otherwise, player *X* can not unscrupulously choose ZD strategies based on player *Y*’s cognition. There is a player *X*’s ZD strategy in player *Y*’s cognition which is not the ZD strategy in player *X*’s cognition. Further, because of the symmetry of $\omega _1$ and $\omega _2$, player *X*’s any available ZD strategy ${\mathbf {p}}$ in $G_2$ is also a ZD strategy in $G_1$ if and only if (1) holds. It indicates that $\Xi (\omega _1)=\Xi (\omega _2)$ and player *X* can choose any ZD strategy based on its own cognition, which does not cause suspicion of the opponent since it is also consistent with player *Y*’s anticipation. Additionally, the slopes of linear relations between players’ utilities may be different, as also shown in Fig. 1, and player *X* can benefit from the misperception by choosing a ZD strategy to improve the corresponding slope.

In fact, (1) covers the following two cases:

1. $2P_i=T_i+S_i$, $i\in \{1,2\}$, is a sufficient condition of (1). Thus, when $2P_i=T_i+S_i$, $i\in \{1,2\}$, player *X*’s any ZD strategy ${\mathbf {p}}$ in $G_1$ is also a ZD strategy in $G_2$. Actually, $2P_i=T_i+S_i$, $i\in \{1,2\}$, means that the sum of players’ utilities when players mutual defect is equal to that when only one player chooses defective strategies.
2. $R_i+P_i=T_i+S_i$, $i\in \{1,2\}$, is another sufficient condition of (1). Thus, when $R_i+P_i=T_i+S_i$, $i\in \{1,2\}$, player *X*’s any ZD strategy ${\mathbf {p}}$ in $G_1$ is also a ZD strategy in $G_2$. Actually, $R_i+P_i=T_i+S_i$, $i\in \{1,2\}$, means that the game has a balanced structure in utilities^32^. At this point, the relationship between cooperation rate and efficiency is monotonous, *i.e.*, the higher the cooperation rate of both sides, the greater the efficiency (the sum of players’ utilities).

Furthermore, for the three special ZD strategies, player *X* can also maintain a linear relationship between players’ utilities in the IPD game with misperception.

#### Equalizer strategy

By choosing equalizer strategies according to player *Y*’s cognition, player *X* can unilaterally set player *Y*’s utilities, as shown in the following corollary.

##### Corollary 1

Player *X*’s any equalizer strategy ${\mathbf {p}}$ in $G_1$ is also an equalizer strategy in $G_2$ if and only if

2 $$\begin{aligned} \frac{R_1-P_1}{R_2-P_2}=\frac{R_1-T_1}{R_2-T_2}=\frac{R_1-S_1}{R_2-S_2}. \end{aligned}$$

(2) is also a sufficient condition of (1). If (2) holds, player *X* can unilaterally set player *Y*’s utility by choosing any equalizer strategy in $G_1$ even though they have different cognitions; otherwise, player *X* can not unscrupulously choose an equalizer strategy based on player *Y*’s cognition since it may not be an equalizer strategy in player *X*’s cognition.

#### Extortion strategy

By choosing extortion strategies according to player *Y*’s cognition, player *X* can get an extortionate share, as shown in the following corollary.

##### Corollary 2

For player *X*’s extortion strategy ${\mathbf {p}}$ with extortion factor $\chi >1$ in $G_1$, ${\mathbf {p}}$ is also an extortion strategy in $G_2$ if (1) and the following inequality hold:

3 $$\begin{aligned} \begin{aligned} (S_1-P_1)(R_2-P_2)-(R_1-P_1)(T_2-P_2)-\chi ((T_1-P_1)(R_2-P_2)-(R_1-P_1)(T_2-P_2))<0. \end{aligned} \end{aligned}$$

Player *X*’s extortion strategy in $G_1$, whose extortion factor $\chi$ satisfies (3), can also ensure that player *X*’s utility is not lower than the opponent’s utility in its own cognition. Thus, player *X* chooses a strategy that satisfies (3), and can also enforce an extortionate share even if there exists misperception.

**Figure 2:**
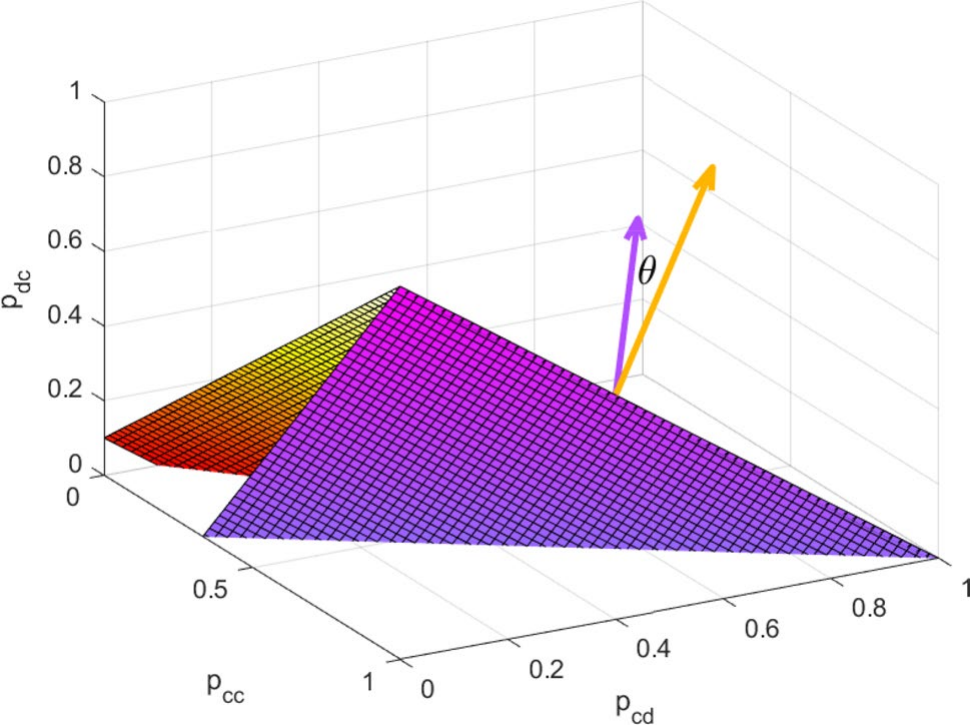
The form of $\theta$ in the IPD game with misperception. Consider $\omega _1=[T_1,R_1,P_1,S_1]=[5,3,1,0]$ and $\omega _2=[T_2,R_2,P_2,S_2]=[\frac{13}{2},6,1,0]$. Suppose $p_{dd}=0$ since it does not influence nonzero canonical angles. The purple (yellow) plane is the available ZD strategy set in $G_1$ ($G_2$) and the purple (yellow) vector is its normal vector. Clearly, $\theta$ is the angle between two normal vectors, which is also the nonzero canonical angle between the available ZD strategy set of $G_1$ and it of $G_2$.

#### Generous strategy

By choosing generous strategies according to player *Y*’s cognition, player *X* may also dominate in the game, as reported in the following corollary.

##### Corollary 3

For player *X*’s generous strategy ${\mathbf {p}}$ with generous factor $\chi >1$ in $G_1$, ${\mathbf {p}}$ is also a generous strategy in $G_2$ if (1) and the following inequality hold:

4 $$\begin{aligned} \begin{aligned}(S_1-R_1)(R_2-P_2)-(R_1-P_1)(T_2-R_2)-\chi ((T_1-R_1)(R_2-P_2)-(R_1-P_1)(T_2-R_2))<0. \end{aligned}\end{aligned}$$

A generous strategy ensures that the utility of the player with generous strategies is not higher than the opponent’s utility, but the player dominants in evolving games^14,33^. Thus, player *X*’s generous strategy, whose generous factor $\chi$ satisfies (4) based on *Y*’s anticipation, can also dominate in the game in player *X*’s cognition. It is rational for player *X* to choose generous strategies which satisfy (4) since the misperception does not change their dominant positions.

**Figure 3:**
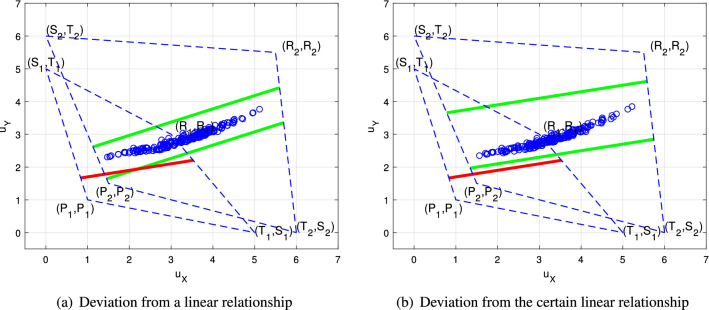
The relation between bounds of Theorems 2 and 3 and players’ utilities in $G_2$. Consider $\omega _1=[T_1,R_1,P_1,S_1]=[5,3,1,0]$ and $\omega _2=[T_2,R_2,P_2,S_2]=[6,\frac{11}{2},\frac{3}{2},0]$. Choose ${\mathbf {p}}=\alpha {\mathbf {S}}^{\omega _1}_{X} +\beta {\mathbf {S}}^{\omega _1}_Y +\gamma {\mathbf {1}}+{\mathbf {p}}_0$, where $(\alpha ,\beta ,\gamma )=(\frac{1}{30},-\frac{1}{6},\frac{1}{4})$, and $(\alpha ',\beta ',\gamma ')=(\frac{38}{165},-\frac{94}{165},\frac{151}{165})$. The red lines describe the relationship between players’ utilities in $G_1$. The green lines describe the bounds according to Theorems 2 and 3. Then we randomly generate 200 player *Y*’s strategies, and the blue circles are $(u^{\omega _2}_X,u^{\omega _2}_Y)$, correspondingly.

### Deviation from misperception

The misperception can lead to a bounded deviation from a linear relationship between players’ expected utilities in player *X*’s cognition. Actually, player *X* chooses a ZD strategy to avoid player *Y*’s suspicion, but player *X* may not enforce a linear relationship between players’ expected utilities in its own cognition. The deviation of the utilities’ relationship is helpful for the player to implement strategies. On the one hand, players’ utilities with misperception go with a bounded deviation from a linear relationship in player *X*’s cognition. Let $\theta$ be the nonzero canonical angles^34^ between the two available ZD strategy sets of $G_1$ and $G_2$, as shown in Fig. 2, and we get the following theorem.

#### Theorem 2

For any player *X*’s ZD strategy ${\mathbf {p}}=\alpha {\mathbf {S}}^{\omega _1}_{X} +\beta {\mathbf {S}}^{\omega _1}_Y +\gamma {\mathbf {1}}+{\mathbf {p}}_0$ in $G_1$, there is $\alpha ', \beta ', \gamma '$ such that

$$\begin{aligned} |\alpha ' u_X^{\omega _2}({\mathbf {p}},{\mathbf {q}})+\beta ' u_Y^{\omega _2}({\mathbf {p}},{\mathbf {q}})+\gamma '|\leqslant ||{\mathbf {p}}||_2\frac{||L_2||_\infty }{||L_2||_2} sin\theta , \forall {\mathbf {q}}, \end{aligned}$$

where $\parallel \cdot \parallel _2$ is the $l_2$ norm, $\parallel \cdot \parallel _\infty$ is the $l_\infty$ norm, and

$$\begin{aligned} \begin{aligned} \theta =&arccos \frac{L_1^TL_2}{||L_1||_2||L_2||_2}, \ L_i=[2P_i-S_i-T_i,R_i-P_i,R_i-P_i,T_i+S_i-2R_i]^T,\ i\in \{1,2\}. \end{aligned} \end{aligned}$$

Misperception makes players’ utilities a bounded deviation from a linear relationship in player *X*’s cognition, that is, $\alpha ' u_X+\beta ' u_Y+\gamma '=0$, even though it is not maintained by choosing ZD strategies in $G_1$, as shown in Fig. 3a. By recognizing the difference between $\omega _1$ and $\omega _2$, player *X* is able to calculate bounds of players’ utility deviation from misperception.

On the other hand, for a given strategy, the deviation from the corresponding linear relationship is also important, while Theorem 2 focuses on the deviation from an existent linear relationship in player *X*’s cognition. The misperception can also bring players’ utilities a bounded deviation from the corresponding linear relationship of the ZD strategy in player *X*’s cognition.

#### Theorem 3

For player *X*’s ZD strategy ${\mathbf {p}}=\alpha {\mathbf {S}}^{\omega _1}_{X} +\beta {\mathbf {S}}^{\omega _1}_Y +\gamma {\mathbf {1}}+{\mathbf {p}}_0$ in $G_1$, the following inequality holds in $G_2$,

$$\begin{aligned} \min (\Gamma )\leqslant \alpha u_X^{\omega _2}({\mathbf {p}},{\mathbf {q}})+\beta u_Y^{\omega _2}({\mathbf {p}},{\mathbf {q}})+\gamma \leqslant \max (\Gamma ), \end{aligned}$$

where

$$\begin{aligned} \Gamma =\{(\alpha +\beta )(R_2-R_1),\alpha (S_2-S_1)+\beta (T_2-T_1),\alpha (T_2-T_1)+\beta (S_2-S_1),(\alpha +\beta )(P_2-P_1)\}. \end{aligned}$$

Any ZD strategy of player *X* based on player *Y*’s cognition can enforce players’ utilities subjected to a bounded deviation from the corresponding linear relationship in player *X*’s cognition, as shown in Fig. 3b. With a ZD strategy ${\mathbf {p}}=\alpha {\mathbf {S}}^{\omega _1}_{X} +\beta {\mathbf {S}}^{\omega _1}_Y +\gamma {\mathbf {1}}+{\mathbf {p}}_0$, player *X* enforces a linear relationship in $G_1$, *i.e.*, $\alpha u_X^{\omega _1}({\mathbf {p}},{\mathbf {q}})+\beta u_Y^{\omega _1}({\mathbf {p}},{\mathbf {q}})+\gamma =0$. Since players’ utilites are $u_X^{\omega _2}$ and $u_Y^{\omega _2}$ in $G_2$, $(u_X^{\omega _2},u_Y^{\omega _2})$ has a bounded deviation from the corresponding relationship $\alpha u_X^{\omega _2}({\mathbf {p}},{\mathbf {q}})+\beta u_Y^{\omega _2}({\mathbf {p}},{\mathbf {q}})+\gamma$.

### Benefit from misperception

**Figure 4:**
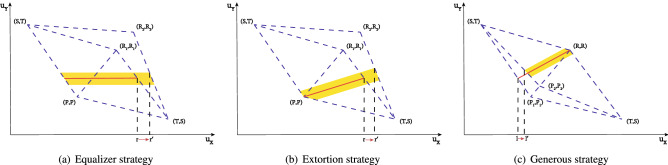
Player *X* can use either equalizer strategies and extortion strategies to raise the supremum of its expected utility or generous strategies to raise the infimum of its expected utility. (**a**) and (**b**) consider that $\omega _1=[T,R_1,P,S]$ and $\omega _2=[T,R_2,P,S]$, where $R_1\ne R_2$; (**c**) considers that $\omega _1=[T,R,P_1,S]$ and $\omega _2=[T,R,P_2,S]$, where $P_1\ne P_2$. The red lines in (**a**), (**b**), and (**c**) describe utilities’ relationships when player *X* chooses an equalizer strategy, an extortion strategy, and a generous strategy in $G_1$, respectively; The yellow area contains all possible relationships between players’ utilities in $G_2$ if player *X* does not change its strategy. In (**a**) and (**b**), *r* is the supremum of player *X*’s utility in $G_1$, and $r'$ is lower than the supremum of player *X*’s utility in $G_2$; In (**c**), *l* is the infimum of player *X*’s utility in $G_1$, and $l'$ is lower than the infimum of player *X*’s utility in $G_2$.

Player *X* is able to take advantage of the misperception since it knows player *Y*’s cognition. To be specific, in IPD without misperception, for any fixed player *X*’s ZD strategy, its utility is influenced by the opponent’s strategy and is always in a closed interval. Player *X* can benefit from the misperception by choosing the strategy, which increases the supremum or the infimum of its own utility in IPD with misperception. Besides, for the three special ZD strategies, player *X*’s ability to improve the supremum/infimum of its own expected utility is shown in Fig. 4, and the following results show how player *X* chooses beneficial strategies.

#### Equalizer strategy

By choosing equalizer strategies according to player *Y*’s cognition, player *X* can improve the supremum of its expected utility.

##### Corollary 4

For player *X*’s equalizer strategy ${\mathbf {p}}=\beta {\mathbf {S}}^{\omega _1}_{Y}+\gamma {\mathbf {1}}+{\mathbf {p}}_0,\beta \ne 0$, in $G_1$, the supremum of player *X*’s expected utility in $G_2$ is larger than that in $G_1$, if

5 $$\begin{aligned} \begin{aligned} a^1_i \frac{\gamma }{\beta }>b^1_i, i\in \{1,2\}, \end{aligned} \end{aligned}$$

where $a^1_i$ and $b^1_i,i\in \{1,2\}$ are parameters shown in “Notations”.

Actually, when player *Y* chooses the always cooperate (ALLC) strategy^35^, *i.e.*, ${\mathbf {q}}=[1,1,1,1]^T$, player *X* gets the supremum of the expected utility in $G_1$ and player *X*’s utility is improved in the IPD game with misperception.

#### Extortion strategy

By choosing extortion strategies according to player *Y*’s cognition, player *X* can also improve the supremum of its expected utility.

##### Corollary 5

For player *X*’s extortion strategy ${\mathbf {p}}$ with extortion factor $\chi >1$ in $G_1$, the supremum of player *X*’s expected utility in $G_2$ is larger than that in $G_1$ if

6 $$\begin{aligned} \begin{aligned}a^2_i\chi ^2+b^2_i\chi +c^2_i<0,i\in \{1,2\}, \end{aligned}\end{aligned}$$

where $a^2_i,b^2_i$, and $c^2_i, i\in \{1,2\}$ are parameters shown in “Notations”.

If player *Y* aims to maximize its own utility with great eagerness, player *Y* chooses the ALLC strategy when player *X* chooses extortion strategies^7^. In this case, by choosing the extortion strategy which satisfies (6), player *X* gets the supremum of the expected utility in $G_1$, where player *X*’s utility is improved in the IPD game with misperception.

#### Generous strategy

By choosing generous strategies according to player *Y*’s cognition, player *X* can also improve the infimum of its expected utility.

##### Corollary 6

For player *X*’s generous strategy ${\mathbf {p}}$ where $\chi >1$, the infimum of player *X*’s expected utility in $G_2$ is larger than that in $G_1$ if

7 $$\begin{aligned} \begin{aligned}a^3_i\chi ^2+b^3_i\chi +c^3_i<0,i\in \{1,2\}, \end{aligned}\end{aligned}$$

where $a^3_i,b^3_i$, and $c^3_i,i\in \{1,2\}$ are parameters shown in “Notations”.

When player *X* chooses generous strategies, player *Y* may choose the always defect (ALLD) strategy^35^, *i.e.*, ${\mathbf {q}}=[0,0,0,0]^T$, which is the worst situation for player *X* since it gets the minimum expected utility in $G_1$. In this case, player *X* is able to improve its expected utility in the worst situation.

## Discussion

This paper concentrates on how misperception affects ZD strategies in IPD games. In our problem, player *Y* is unaware of the different cognitions, but it believes that player *X* takes a ZD strategy, while player *X* can detect the misperception. Since each player observes the strategy in sequence, to avoid player *Y*’s suspicion, player *X* needs to keep its ZD strategies. Therefore, we have explored the ZD strategies in IPD with misperception—a linear relationship between the two players’ expected utilities. In fact, under this affine constraint, player *X* can ignore the misperception and choose ZD strategies freely. Specifically, we have studied the three typical ZD strategies—equalizer, extortion, and generous ones, and moreover, we have investigated the players’ expected utility deviation from misperception in player *X*’s cognition. For clarification, we have described the deviation not only from the corresponding linear relationship of the ZD strategy but also from another linear relationship that is not directly obtained by player *X*. Finally, we have revealed that the player equipped with ZD strategies may benefit from misperception to improve its own utility. Thus, player *X* can adopt special equalizer, extortion, or generous strategies to promote the supremum/infimum of its utility in IPD with misperception.

Although both Fig. 3a, b illustrates the players’ utilitiy deviation, they are actually derived from different perspectives. Figure 3a describes the deviation from a linear relationship, that is, $\alpha ' u_X+\beta ' u_Y+\gamma '=0$, where the specific values of $\alpha ', \beta ',\gamma '$ are not given in Theorem 2. It is helpful for player *X* to choose beneficial strategies if aiming to get as close to a linear relationship as possible, but no caring about what the linear relationship is. On the other hand, Fig. 3b indicates that the deviation is derived from a certation linear relation, that is, $\alpha u_X+\beta u_Y+\gamma =0$, where $\alpha ,\beta ,\gamma$ are decided by the given ZD strategy. The deviation bounds, according to Theorem 3, are parallel to the linear relationship of the ZD strategy, which helps us analyze the supremum/infimum of player *X*’s utility with misperception.

Moreover, players may actively adopt misperception to deceive their opponents. For example, players may be able to control their opponents’ observation by interfering with private monitoring^36^, or deliberately mislead their opponents with imitative strategies such as “fake news”^37,38^. In fact, players may change the parameters and utilities of IPD in others’ cognition by deceiving their opponents. Hence, how the player who adopts ZD strategies benefits from deception in IPD without the opponent’s awareness is also worth analyzing. Since the ZD strategy has also been widely applied in other complicated situations, such as non-symmetric games^39^, PGG^10^, and evolutionary situations^33^, the misperception influence analysis will be extended to the ZD strategies in these practical fields.

## Notations

$M=[M_{jk}]_{4\times 4}$ denotes the probability from the last state $k\in \{cc,cd,dc,dd\}$ to the next state $j\in \{cc,cd,dc,dd\}$ in each round, as shown in the following:

$$M=\left[ \begin{array}{llll} p_{cc} q_{cc} & p_{cc}\left( 1-q_{cc}\right) & \left( 1-p_{cc}\right) q_{cc} & \left( 1-p_{cc}\right) \left( 1-q_{cc}\right) \\ p_{cd} q_{dc} & p_{cd}\left( 1-q_{dc}\right) & \left( 1-p_{cd}\right) q_{dc} & \left( 1-p_{cd}\right) \left( 1-q_{dc}\right) \\ p_{dc} q_{cd} & p_{dc}\left( 1-q_{cd}\right) & \left( 1-p_{dc}\right) q_{cd} & \left( 1-p_{dc}\right) \left( 1-q_{cd}\right) \\ p_{dd} q_{dd} & p_{dd}\left( 1-q_{dd}\right) & \left( 1-p_{dd}\right) q_{dd} & \left( 1-p_{dd}\right) \left( 1-q_{dd}\right) \end{array}\right] .$$

Thus, *M* is regular when all elements of *M* are positive, *e.g.*, $0<p_{xy},q_{xy}<1, xy\in \{cc,cd,dc,dd\}$. Denote $$\Upsilon (a,b)=det\left( \begin{array}{cc} a_1&a_2\\ b_1& b_2 \end{array}\right)$$, $$\Lambda (a,b,c,d)=det\left( \begin{array}{cc} a_1&b_2\\ c_1& d_2 \end{array}\right)$$, and $\delta =\max \{|R_2-R_1|,|S_2-S_1|,|T_2-T_1|,|P_2-P_1|\}$. The notations in Corollary 4 are shown as follows:

$$\begin{aligned} a^1_1=&\Upsilon (R-S,T-R),\\ b^1_1=&\Upsilon (R(T-S),R-S) +(R_1-S_1)(T_2-R_2)\delta ,\\ a^1_2=&\Lambda (R-S,T-R,T-R,R-S),\\ b^1_2=&\Lambda (R(T-S),R(T-S),R-S,T-R)+(R_1-S_1)(R_2-S_2)\delta . \end{aligned}$$

The notations in Corollary 5 are shown as follows:

$$\begin{aligned} a^2_1=&\Upsilon (R(T-S),R-S)-\Upsilon (P(T-R),R-S)+\delta (T_2-R_2)(R_1-S_1),\\ b^2_1=&\Upsilon (R(T-S),R-S)-\Upsilon (P(T-R),T+S-2R)+\delta (T_2-R_2)(T_1-S_1),\\ c^2_1=&(P_1-P_2+\delta )(T_2-R_2)(T_1-R_1),\\ a^2_2=&\Lambda (R(T-S),R(T-S),R-S,T-R)-\Lambda (P(T-R),P(R-S),R-S,T-R)+\delta (R_2-S_2)(R_1-S_1),\\ b^2_2=&\Lambda (R(T-S),R(T-S),T-R,R-S)-\Lambda (P(T-R),P(R-S),2R-T-S,T+S-2R)+\delta (R_2\!-\!S_2)(T_1\!-\!S_1),\\ c^2_2=&(P_1-P_2+\delta )(R_2-S_2)(T_1-R_1). \end{aligned}$$

The notations in Corollary 6 are shown as follows:

$$\begin{aligned} a^3_1=&\Upsilon (P(T-S),T-P)-\Upsilon (R(P-S),T-P)+\delta (T_1-P_1)(P_2-S_2),\\ b^3_1=&\Upsilon (P(T-S),P-S)-\Upsilon (R(P-S),2P-T-S)+\delta (T_1-S_1)(P_2-S_2) ,\\ c^3_1=&(R_1-R_2+\delta )(T_1-S_1)(P_2-S_2),\\ a^3_2=&\Lambda (P(T-S),P(T-S),T-P,P-S)-\Lambda (R(P-S),R(T-P),T-P,P-S)+\delta (T_1-P_1)(T_2-P_2),\\ b^3_2=&\Lambda (P(T-S),P(T-S),P-S,T-P)-\Lambda (R(P-S),R(T-P),2P-T-S,T+S-2P) +\delta (T_1\!-\!S_2)(T_2\!-\!P_2),\\ c^3_2=&(R_1-R_2+\delta )(P_1-S_1)(T_2-P_2). \end{aligned}$$

## Supplementary information

Supplementary Information.
