# Supplementary material for: Misperception influence on zero-determinant strategies in iterated Prisoner’s Dilemma
Source: Sci Rep. 2022 Mar 25;12:5174. doi: 10.1038/s41598-022-08750-8 (PMC8956668; doi:10.1038/s41598-022-08750-8)
Supplement: Supplementary file 1 — Supplementary Information. [file 41598_2022_8750_MOESM1_ESM.pdf]

# Supplementary Information: Misperception Influence on Zero-Determinant Strategies in Iterated Prisoner's Dilemma

**Zhaoyang Cheng[1,2], Guanpu Chen[3,1], and Yiguang Hong[4,1,*]**

[1]Key Laboratory of Systems and Control, Academy of Mathematics and Systems Science, Beijing, 100190, China
[2]School of Mathematical Sciences, University of Chinese Academy of Sciences, Beijing, 100190, China
[3]JD Explore Academy, Beijing, China
[4]Department of Control Science and Engineering, Tongji University, Shanghai, 201804, China
[*]yghong@iss.ac.cn

## 1 Proof of Theorem 1

Suppose that any ZD strategy $\mathbf{p}$ of player $X$ in $G_1$ is also a ZD strategy in $G_2$. Then for any $\alpha,\beta,\gamma$, and $\mathbf{p}=\alpha\mathbf{S}_X^{\omega_1}+\beta\mathbf{S}_Y^{\omega_1}+\gamma\mathbf{1}+\mathbf{p}_0$, there exists $\alpha',\beta',\gamma'$ such that $\mathbf{p}=\alpha'\mathbf{S}_X^{\omega_2}+\beta'\mathbf{S}_Y^{\omega_2}+\gamma'\mathbf{1}+\mathbf{p}_0$. Then the following equations has at least one solution:

$$\begin{aligned}\alpha'R_2+\beta'R_2+\gamma' &= \alpha R_1+\beta R_1+\gamma,\\ \alpha'S_2+\beta'T_2+\gamma' &= \alpha S_1+\beta T_1+\gamma,\\ \alpha'T_2+\beta'S_2+\gamma' &= \alpha T_1+\beta S_1+\gamma,\\ \alpha'P_2+\beta'P_2+\gamma' &= \alpha P_1+\beta P_1+\gamma.\end{aligned} \tag{1}$$

Notice that the first three equations of (1) have a solution:

$$\begin{aligned}\alpha' &= \frac{1}{2}\left(\frac{(\alpha+\beta)(2R_1-T_1-S_1)}{2R_2-T_2-S_2}+\frac{(\alpha-\beta)(S_1-T_1)}{S_2-T_2}\right),\\ \beta' &= \frac{1}{2}\left(\frac{(\alpha+\beta)(2R_1-T_1-S_1)}{2R_2-T_2-S_2}-\frac{(\alpha-\beta)(S_1-T_1)}{S_2-T_2}\right),\\ \gamma' &= \alpha R_1+\beta R_1+\gamma-R_2\frac{(\alpha+\beta)(2R_1-T_1-S_1)}{2R_2-T_2-S_2}.\end{aligned} \tag{2}$$

Since $det\begin{bmatrix} R_2 & R_2 & 1\\ S_2 & T_2 & 1\\ T_2 & S_2 & 1\end{bmatrix}=(T_2-S_2)(2R_2-S_2-T_2)\neq 0$, (2) is the unique solution of the first three equations of (1) . Then

$$\begin{aligned}0 =& \alpha'P_2+\beta'P_2+\gamma'-\alpha P_1+\beta P_1+\gamma,\\ =&\frac{(\alpha+\beta)(2R_1-T_1-S_1)}{2R_2-T_2-S_2}P_2+\alpha R_1+\beta R_1+\gamma-R_2\frac{(\alpha+\beta)(2R_1-T_1-S_1)}{2R_2-T_2-S_2}-\alpha P_1+\beta P_1+\gamma\\ =&\alpha\left(\frac{2R_1-T_1-S_1}{2R_2-T_2-S_2}(P_2-R_2)+(R_1-P_1)\right)+\beta\alpha\left(\frac{2R_1-T_1-S_1}{2R_2-T_2-S_2}(P_2-R_2)+(R_1-P_1)\right).\end{aligned}$$

By the arbitrariness of $\alpha,\beta,\gamma$,

$$\frac{2R_1-T_1-S_1}{2R_2-T_2-S_2}(P_2-R_2)+(R_1-P_1)=0. \tag{3}$$

Then (3) is equivalent to

$$\frac{R_1-P_1}{2R_1-S_1-T_1}=\frac{R_2-P_2}{2R_2-S_2-T_2}.$$

On the contrary, suppose that $\frac{R_1-P_1}{2R_1-S_1-T_1}=\frac{R_2-P_2}{2R_2-S_2-T_2}$. For any $\alpha,\beta,\gamma$, and $\mathbf{p}=\alpha\mathbf{S}_X^{\omega_1}+\beta\mathbf{S}_Y^{\omega_1}+\gamma\mathbf{1}+\mathbf{p}_0$, take $\alpha',\beta',\gamma'$ as (2). Then (1) holds if $\frac{R_1-P_1}{2R_1-S_1-T_1}=\frac{R_2-P_2}{2R_2-S_2-T_2}$. Thus, any ZD strategy $\mathbf{p}$ of player $X$ in $G_1$ is also a ZD strategy in $G_2$.

## 2 Proof of Corollary 1

Suppose that player $X$'s any equalizer strategy $\mathbf{p}$ in $G_1$ is also an equalizer strategy in $G_2$. Then for any $\beta,\gamma$, and $\mathbf{p} = \beta\mathbf{S}_Y^{\omega_1} + \gamma\mathbf{1} + \mathbf{p}_0$, there exists $\beta',\gamma'$ such that $\mathbf{p} = \beta'\mathbf{S}_Y^{\omega_2} + \gamma'\mathbf{1} + \mathbf{p}_0$. Then the following equations has at least one solution:

$$\begin{aligned} \beta' R_2 + \gamma' &= \beta R_1 + \gamma, \\ \beta' T_2 + \gamma' &= \beta T_1 + \gamma, \\ \beta' S_2 + \gamma' &= \beta S_1 + \gamma, \\ \beta' P_2 + \gamma' &= \beta P_1 + \gamma. \end{aligned} \tag{4}$$

The first two equations of (4) have a solution:

$$\begin{aligned} \beta' &= \beta\frac{R_1 - T_1}{R_2 - T_2}, \\ \gamma' &= \beta R_1 + \gamma - \beta R_2\frac{R_1 - T_1}{R_2 - T_2}. \end{aligned} \tag{5}$$

Since $det\begin{bmatrix} R_2 & 1 \\ T_2 & 1 \end{bmatrix} = R_2 - T_2 \neq 0$, (5) is the unique solution of the first two equations of (4). Then the last two equations of (4) are converted to:

$$\begin{aligned} \beta\frac{R_1 - T_1}{R_2 - T_2} &= \beta\frac{R_1 - S_1}{R_2 - S_2}, \\ \beta\frac{R_1 - T_1}{R_2 - T_2} &= \beta\frac{R_1 - P_1}{R_2 - P_2}. \end{aligned} \tag{6}$$

By the arbitrariness of $\beta,\gamma$, we have

$$\frac{R_1 - P_1}{R_2 - P_2} = \frac{R_1 - T_1}{R_2 - T_2} = \frac{R_1 - S_1}{R_2 - S_2}.$$

On the contrary, suppose that $\frac{R_1-P_1}{R_2-P_2} = \frac{R_1-T_1}{R_2-T_2} = \frac{R_1-S_1}{R_2-S_2}$. For any $\beta,\gamma$, and $\mathbf{p} = \beta\mathbf{S}_Y^{\omega_1} + \gamma\mathbf{1} + \mathbf{p}_0$, take $\beta',\gamma'$ as (5). (4) holds if $\frac{R_1-P_1}{R_2-P_2} = \frac{R_1-T_1}{R_2-T_2} = \frac{R_1-S_1}{R_2-S_2}$. Thus, player $X$'s any equalizer strategy $\mathbf{p}$ in $G_1$ is also an equalizer strategy in $G_2$.

## 3 Proof of Corollary 2

For any $\phi,\chi > 1$, and $\mathbf{p} = \phi[(\mathbf{S}_X^{\omega_1} - P_1\mathbf{1}) - \chi(\mathbf{S}_Y^{\omega_1} - P_1\mathbf{1})] + \mathbf{p}_0$, we want to find if there exists $\phi',\chi'$ such that

$$\mathbf{p} = \phi'[(\mathbf{S}_X^{\omega_2} - P_2\mathbf{1}) - \chi'(\mathbf{S}_Y^{\omega_2} - P_2\mathbf{1})] + \mathbf{p}_0, \chi' \geqslant 1. \tag{7}$$

(7) is equivalent to the following equations:

$$\begin{aligned} \phi'[(R_2 - P_2) - \chi'(R_2 - P_2)] &= \phi[(R_1 - P_1) - \chi(R_1 - P_1)], \\ \phi'[(S_2 - P_2) - \chi'(T_2 - P_2)] &= \phi[(S_1 - P_1) - \chi(T_1 - P_1)], \\ \phi'[(T_2 - P_2) - \chi'(S_2 - P_2)] &= \phi[(T_1 - P_1) - \chi(S_1 - P_1)]. \end{aligned} \tag{8}$$

If $\phi \neq 0$, we have $\phi' \neq 0$. The first two equations of (8) have a solution:

$$\begin{aligned} \phi' &= \frac{\phi[(S_1 - P_1) - \chi(T_1 - P_1)](R_2 - P_2) - \phi[(R_1 - P_1) - \chi(R_1 - P_1)](T_2 - P_2)}{(R_2 - P_2)(S_2 - T_2)}, \\ \chi' &= 1 - \frac{\phi[(R_1 - P_1) - \chi(R_1 - P_1)]}{\phi'(R_2 - P_2)}. \end{aligned} \tag{9}$$

Then the last equation of (8) is converted to:

$$\begin{aligned} &\phi'[(T_2 - P_2) - \chi'(S_2 - P_2)] - \phi[(T_1 - P_1) - \chi(S_1 - P_1)] \\ =&\phi'\left[(T_2 - P_2) - \left(1 - \frac{\phi[(R_1 - P_1) - \chi(R_1 - P_1)]}{\phi'(R_2 - P_2)}\right)(S_2 - P_2)\right] - \phi[(T_1 - P_1) - \chi(S_1 - P_1)] \\ =&\phi'(T_2 - S_2) + \frac{1}{R_2 - P_2}\phi[(R_1 - P_1)(S_2 - P_2) - \chi(R_1 - P_1)(S_2 - P_2) - (T_1 - P_1)(R_2 - P_2) + \chi(S_1 - P_1)(R_2 - P_2)] \\ =&\frac{\phi}{R_2 - P_2}[-(S_1 + T_1 - 2P_1)(R_2 - P_2) + (R_1 - P_1)(T_2 + S_2 - 2P_2) + \chi(T_1 + S_1 - 2P_1)(R_2 - P_2) - \chi(R_1 - P_1)(T_2 + S_2 - P_2))] \\ =&\frac{\phi}{R_2 - P_2}[(1 - \chi)((R_1 - P_1)(T_2 + S_2 - 2P_2) - (S_1 + T_1 - 2P_1)(R_2 - P_2))] \end{aligned}$$

Thus, $\phi'[(T_2-P_2)-\chi'(S_2-P_2)]-\phi[(T_1-P_1)-\chi(S_1-P_1)]=0$ if $\frac{R_1-P_1}{2R_1-S_1-T_1}=\frac{R_2-P_2}{2R_2-S_2-T_2}$. Besides,

$$\begin{aligned}\chi' =&1-\frac{[(R_1-P_1)-\chi(R_1-P_1)](S_2-T_2)}{[(S_1-P_1)-\chi(T_1-P_1)](R_2-P_2)-[(R_1-P_1)-\chi(R_1-P_1)](T_2-P_2)}\\ =&1-\frac{(1-\chi)(R_1-P_1)(S_2-T_2)}{(S_1-P_1)(R_2-P_2)-(R_1-P_1)(T_2-P_2)-\chi((T_1-P_1)(R_2-P_2)-(R_1-P_1)(T_2-P_2))}\end{aligned}$$

Since $(S_1-P_1)(R_2-P_2)-(R_1-P_1)(T_2-P_2)-\chi((T_1-P_1)(R_2-P_2)-(R_1-P_1)(T_2-P_2))<0$, we have $\chi'>1$. Actually, if $\phi=0$, we have $\phi'=0$ and $\mathbf{p}=\mathbf{p}_0=[1,1,0,0]^T$. Then $\mathbf{p}$ is also an extortion strategy in $G_2$.

## 4 Proof of Corollary 3

For any $\phi,\chi>1$, and $\mathbf{p}=\phi[(\mathbf{S}_X^{\omega_1}-R_1\mathbf{1})-\chi(\mathbf{S}_Y^{\omega_1}-R_1\mathbf{1})]$, we want to find if there exists $\phi',\chi'$ such that

$$\phi'[(\mathbf{S}_X^{\omega_2}-R_2\mathbf{1})-\chi'(\mathbf{S}_Y^{\omega_2}-R_2\mathbf{1})]=\mathbf{p}. \tag{10}$$

(10) is equivalent to the following equations:

$$\begin{aligned}\phi'[(P_2-R_2)-\chi'(P_2-R_2)]&=\phi[(P_1-R_1)-\chi(P_1-R_1)],\\ \phi'[(S_2-R_2)-\chi'(T_2-R_2)]&=\phi[(S_1-R_1)-\chi(T_1-R_1)],\\ \phi'[(T_2-R_2)-\chi'(S_2-R_2)]&=\phi[(T_1-R_1)-\chi(S_1-R_1)].\end{aligned} \tag{11}$$

Similar to the proof of Corollary 2, if $\phi\neq 0$, the first two equations of (12) have a solution:

$$\begin{aligned}\phi' =&\frac{\phi[(S_1-R_1)-\chi(T_1-R_1)](R_2-P_2)-\phi[(R_1-P_1)-\chi(R_1-P_1)](T_2-R_2)}{(R_2-P_2)(S_2-T_2)},\\ \chi' =&1-\frac{\phi[(R_1-P_1)-\chi(R_1-P_1)]}{\phi'(R_2-P_2)}.\end{aligned} \tag{12}$$

Also, $\phi'[(T_2-R_2)-\chi'(S_2-R_2)]=\phi[(T_1-R_1)-\chi(S_1-R_1)]$ if $\frac{R_1-P_1}{2R_1-S_1-T_1}=\frac{R_2-P_2}{2R_2-S_2-T_2}$. Besides, we also have

$$\chi' =1-\frac{(1-\chi)(R_1-P_1)(S_2-T_2)}{(S_1-R_1)(R_2-P_2)-(R_1-P_1)(T_2-R_2)-\chi((T_1-R_1)(R_2-P_2)-(R_1-P_1)(T_2-R_2))}$$

If $(S_1-R_1)(R_2-P_2)-(R_1-P_1)(T_2-R_2)-\chi((T_1-R_1)(R_2-P_2)-(R_1-P_1)(T_2-R_2))<0$, we have $\chi'>1$. Thus, $\mathbf{p}$ is an extortion strategy in $G_2$ when $\phi\neq 0$. Actually, if $\phi=0$, we have $\phi'=0$ and $\mathbf{p}=\mathbf{p}_0=[1,1,0,0]^T$. Then $\mathbf{p}$ is also a generous strategy in $G_2$.

## 5 Proof of Theorem 2

$S_X^{\omega_i}$, $S_Y^{\omega_i}$, and $\mathbf{1}$ are orthogonal since $det\begin{bmatrix}R_i & R_i & 1\\ S_i & T_i & 1\\ T_i & S_i & 1\end{bmatrix}=(T_i-S_i)(2R_i-S_i-T_i)\neq 0$. Denote $\Phi_i$ as the space spanned by $S_X^{\omega_i}$, $S_Y^{\omega_i}$, and $\mathbf{1}$. Consider $L_i=[2P_i-S_i-T_i,R_i-P_i,R_i-P_i,T_i+S_i-2R_i]^T$. Then we have

$$\begin{aligned}L_i^T S_X^{\omega_i}&=(2P_i-S_i-T_i)R_i+(R_i-P_i)S_i+(R_i-P_i)T_i+(T_i+S_i-2R_i)P_i=0,\\ L_i^T S_Y^{\omega_i}&=(2P_i-S_i-T_i)R_i+(R_i-P_i)T_i+(R_i-P_i)S_i+(T_i+S_i-2R_i)P_i=0,\\ L_i^T\mathbf{1}&=(2P_i-S_i-T_i)+(R_i-P_i)+(R_i-P_i)+(T_i+S_i-2R_i)=0.\end{aligned}$$

Also, $||L_i||_2^2=(2P_i-S_i-T_i)^2+2(R_i-P_i)^2+(T_i+S_i-2R_i)^2>0$. Thus, $S_X^{\omega_i}$, $S_Y^{\omega_i}$, $\mathbf{1}$, and $L_2$ are orthogonal in $\mathbb{R}^4$, and $L_i$ is orthogonal to $\Phi_i$. According to Press and Dyson[1], the nonzero canonical angles between $\phi_1$ and $\phi_2$ is

$$\theta =arccos\frac{L_1^T L_2}{||L_1||_2||L_2||_2}.$$

For any $\alpha,\beta,\gamma$, there exists $\alpha',\beta',\gamma'$ such that $\alpha\mathbf{S}_X^{\omega_1}+\beta\mathbf{S}_Y^{\omega_1}+\gamma\mathbf{1}=\alpha'\mathbf{S}_X^{\omega_2}+\beta'\mathbf{S}_Y^{\omega_2}+\gamma'\mathbf{1}+\xi\frac{L_2}{||L_2||_2}$, where $\xi=||\mathbf{p}||_2 sin\theta$ is the norm of the projection from $\alpha\mathbf{S}_X^{\omega_1}+\beta\mathbf{S}_Y^{\omega_1}+\gamma\mathbf{1}$ to $\frac{L_2}{||L_2||_2}$.

For any $\mathbf{f}=[f_1,f_2,f_3,f_4]^T\in\mathbb{R}^4, \mathbf{p}'=[p'_{cc},p'_{cd},p'_{dc},p'_{dd}]^T, \mathbf{q}'=[q'_{cc},q'_{dc},q'_{cd},q'_{dd}]^T$, let

$$D(\mathbf{p}',\mathbf{q}',\mathbf{f})=det\begin{bmatrix} p'_{cc}q'_{cc}-1 & p'_{cc}-1 & q'_{cc}-1 & f_1\\ p'_{cd}q'_{dc} & p'_{cd}-1 & q'_{dc} & f_2\\ p'_{dc}q'_{cd} & p'_{dc} & q'_{cd}-1 & f_3\\ p'_{dd}q'_{dd} & p'_{dd} & q'_{dd} & f_4\end{bmatrix}.$$

According to Press and Dyson[1],

$$\alpha' u_X^{\omega_2}(\mathbf{p},\mathbf{q})+\beta' u_Y^{\omega_2}(\mathbf{p},\mathbf{q})+\gamma' =\frac{D(\mathbf{p},\mathbf{q},\alpha'\mathbf{S}_X^{\omega_2}+\beta'\mathbf{S}_Y^{\omega_2}+\gamma'\mathbf{1})}{D(\mathbf{p},\mathbf{q},\mathbf{1})},$$

Then

$$\begin{aligned}D(\mathbf{p},\mathbf{q},\alpha'\mathbf{S}_X^{\omega_2}+\beta'\mathbf{S}_Y^{\omega_2}+\gamma'\mathbf{1}) =&det\begin{bmatrix} p_{cc}q_{cc}-1 & \alpha R_1+\beta R_1+\gamma & q_{cc}-1 & \alpha' R_2+\beta' R_2+\gamma'\\ p_{cd}q_{dc} & \alpha S_1+\beta T_1+\gamma & q_{dc} & \alpha' S_2+\beta' T_2+\gamma'\\ p_{dc}q_{cd} & \alpha T_1+\beta S_1+\gamma & q_{cd}-1 & \alpha' T_2+\beta' S_2+\gamma'\\ p_{dd}q_{dd} & \alpha P_1+\beta P_1+\gamma & q_{dd} & \alpha' P_2+\beta' P_2+\gamma'\end{bmatrix}\\
=&det\begin{bmatrix} p_{cc}q_{cc}-1 & \alpha R_1+\beta R_1+\gamma & q_{cc}-1 & \xi\frac{1}{||L_2||_2}(2P_2-S_2-T_2)\\ p_{cd}q_{dc} & \alpha S_1+\beta T_1+\gamma & q_{dc} & \xi\frac{1}{||L_2||_2}(R_2-P_2)\\ p_{dc}q_{cd} & \alpha T_1+\beta S_1+\gamma & q_{cd}-1 & \xi\frac{1}{||L_2||_2}(R_2-P_2)\\ p_{dd}q_{dd} & \alpha P_1+\beta P_1+\gamma & q_{dd} & \xi\frac{1}{||L_2||_2}(T_2+S_2-2R_2)\end{bmatrix}\\
=&\xi\frac{1}{||L_2||_2}det\begin{bmatrix} p_{cc}q_{cc}-1 & \alpha R_1+\beta R_1+\gamma & q_{cc}-1 & 2P_2-S_2-T_2\\ p_{cd}q_{dc} & \alpha S_1+\beta T_1+\gamma & q_{dc} & R_2-P_2\\ p_{dc}q_{cd} & \alpha T_1+\beta S_1+\gamma & q_{cd}-1 & R_2-P_2\\ p_{dd}q_{dd} & \alpha P_1+\beta P_1+\gamma & q_{dd} & T_2+S_2-2R_2\end{bmatrix}\\
=&||\mathbf{p}||_2 sin\theta\frac{1}{||L_2||_2}D(\mathbf{p},\mathbf{q},L_2).\end{aligned}$$

Thus,

$$\alpha' u_X^{\omega_2}(\mathbf{p},\mathbf{q})+\beta' u_Y^{\omega_2}(\mathbf{p},\mathbf{q})+\gamma' = ||\mathbf{p}||_2 sin\theta\frac{1}{||L_2||_2}\frac{D(\mathbf{p},\mathbf{q},L_2)}{D(\mathbf{p},\mathbf{q},\mathbf{1})}.$$

Actually,

$$\frac{D(\mathbf{p},\mathbf{q},L_2)}{D(\mathbf{p},\mathbf{q},\mathbf{1})}=v_{cc}(2P_2-S_2-T_2)+v_{cd}(R_2-P_2)+v_{dc}(R_2-P_2)+v_{dd}(T_2+S_2-2R_2),$$

where

$$v_{cc}=-\frac{1}{D(\mathbf{p},\mathbf{q},\mathbf{1})}det\begin{bmatrix} p_{cd}q_{dc} & p_{cd}-1 & q_{dc}\\ p_{dc}q_{cd} & p_{dc} & q_{cd}-1\\ p_{dd}q_{dd} & p_{dd} & q_{dd}\end{bmatrix},\quad v_{cd}=\frac{1}{D(\mathbf{p},\mathbf{q},\mathbf{1})}det\begin{bmatrix} p_{cc}q_{cc}-1 & p_{cc}-1 & q_{cc}-1\\ p_{dc}q_{cd} & p_{dc} & q_{cd}-1\\ p_{dd}q_{dd} & p_{dd} & q_{dd}\end{bmatrix},$$

$$v_{dc}=-\frac{1}{D(\mathbf{p},\mathbf{q},\mathbf{1})}det\begin{bmatrix} p_{cc}q_{cc}-1 & p_{cc}-1 & q_{cc}-1\\ p_{cd}q_{dc} & p_{cd}-1 & q_{dc}\\ p_{dd}q_{dd} & p_{dd} & q_{dd}\end{bmatrix},\ v_{dd}=\frac{1}{D(\mathbf{p},\mathbf{q},\mathbf{1})}det\begin{bmatrix} p_{cc}q_{cc}-1 & p_{cc}-1 & q_{cc}-1\\ p_{cd}q_{dc} & p_{cd}-1 & q_{dc}\\ p_{dc}q_{cd} & p_{dc} & q_{cd}-1\end{bmatrix}.$$

According to Press and Dyson[1], $v_{xy}\in[0,1], xy\in\{cc,dc,cd,dd\}$, and $v_{cc}+v_{cd}+v_{dc}+v_{dd}=1$. Then

$$\left|\frac{D(\mathbf{p},\mathbf{q},L_2)}{D(\mathbf{p},\mathbf{q},\mathbf{1})}\right|\leqslant \max\{|2P_2-S_2-T_2|,|R_2-P_2|,|R_2-P_2|,|T_2+S_2-2R_2|\}=||L_2||_\infty.$$

Therefore, $|\alpha' u_X^{\omega_2}(\mathbf{p},\mathbf{q})+\beta' u_Y^{\omega_2}(\mathbf{p},\mathbf{q})+\gamma'|\leqslant ||\mathbf{p}||_2\frac{||L_2||_\infty}{||L_2||_2}sin\theta, \forall\mathbf{q}$.

## 6 Proof of Theorem 3

For any $\alpha,\beta,\gamma$, we have

$$\begin{aligned}&\alpha u_X^{\omega_2}(\mathbf{p},\mathbf{q})+\beta u_Y^{\omega_2}(\mathbf{p},\mathbf{q})+\gamma\\ =&\frac{D(\mathbf{p},\mathbf{q},\alpha\mathbf{S}_X^{\omega_2}+\beta\mathbf{S}_Y^{\omega_2}+\gamma\mathbf{1})}{D(\mathbf{p},\mathbf{q},\mathbf{1})},\end{aligned}$$

Acutally,

$$D(\mathbf{p},\mathbf{q},\alpha'\mathbf{S}_X^{\omega_2}+\beta'\mathbf{S}_Y^{\omega_2}+\gamma'\mathbf{1}) = det\begin{bmatrix} p_{cc}q_{cc}-1 & \alpha R_1+\beta R_1+\gamma & q_{cc}-1 & \alpha R_2+\beta R_2+\gamma \\ p_{cd}q_{dc} & \alpha S_1+\beta T_1+\gamma & q_{dc} & \alpha S_2+\beta T_2+\gamma \\ p_{dc}q_{cd} & \alpha T_1+\beta S_1+\gamma & q_{cd}-1 & \alpha T_2+\beta S_2+\gamma \\ p_{dd}q_{dd} & \alpha P_1+\beta P_1+\gamma & q_{dd} & \alpha P_2+\beta P_2+\gamma \end{bmatrix}$$

$$= det\begin{bmatrix} p_{cc}q_{cc}-1 & \alpha R_1+\beta R_1+\gamma & q_{cc}-1 & (\alpha+\beta)(R_2-R_1) \\ p_{cd}q_{dc} & \alpha S_1+\beta T_1+\gamma & q_{dc} & \alpha(S_2-S_1)+\beta(T_2-T_1) \\ p_{dc}q_{cd} & \alpha T_1+\beta S_1+\gamma & q_{cd}-1 & \alpha(T_2-T_1)+\beta(S_2-S_1) \\ p_{dd}q_{dd} & \alpha P_1+\beta P_1+\gamma & q_{dd} & (\alpha+\beta)(P_2-P_1) \end{bmatrix}.$$

Thus, we have

$$\begin{aligned}&\alpha u_X^{\omega_2}(\mathbf{p},\mathbf{q})+\beta u_Y^{\omega_2}(\mathbf{p},\mathbf{q})+\gamma \\ =&v_{cc}(\alpha+\beta)(R_2-R_1)+v_{cd}(\alpha(S_2-S_1)+\beta(T_2-T_1))+v_{dc}(\alpha(T_2-T_1)+\beta(S_2-S_1))+v_{dd}(\alpha+\beta)(P_2-P_1),\end{aligned}$$

where $v_{xy}, xy\in\{cc,dc,cd,dd\}$ is the same in the proof of Theorem 2. Since $v_{xy}\in[0,1], xy\in\{cc,dc,cd,dd\}$, and $v_{cc}+v_{cd}+v_{dc}+v_{dd}=1$, we have

$$\min(\Gamma)\leqslant \alpha u_X^{\omega_2}(\mathbf{p},\mathbf{q})+\beta u_Y^{\omega_2}(\mathbf{p},\mathbf{q})+\gamma\leqslant\max(\Gamma), \quad (13)$$

where

$$\Gamma=\{(\alpha+\beta)(R_2-R_1),\alpha(S_2-S_1)+\beta(T_2-T_1),\alpha(T_2-T_1)+\beta(S_2-S_1),(\alpha+\beta)(P_2-P_1)\}.$$

## 7 Proof of Corollary 4

When player $Y$ chooses the always cooperate (ALLC) strategy, $(u_X^{\omega_i},u_Y^{\omega_i})$ is on $(R_i-S_i)u_X^{\omega_i}+(T_i-R_i)u_Y^{\omega_i}=R_i(T_i-S_i)$ in $G_i, i\in\{1,2\}$. For any $\beta\neq 0,\gamma$, suppose player $X$ chooses an equalizer strategy $\mathbf{p}=\beta\mathbf{S}_Y^{\omega_1}+\gamma\mathbf{1}+\mathbf{p}_0$ in $G_1$. When player $Y$ choose the ALLC strategy, player $X$ get the supremum of its expected utility, $\bar{u}_X^{\omega_1}$, in $G_1$. $\bar{u}_X^{\omega_1}$ is the solution of the following equations:

$$\begin{cases}\beta u_Y+\gamma=0,\\ (R_1-S_1)u_X+(T_1-R_1)u_Y-R_1(T_1-S_1)=0.\end{cases}$$

Then $\bar{u}_X^{\omega_1}=\frac{R_1(T_1-S_1)+(T_1-R_1)\frac{\gamma}{\beta}}{(R_1-S_1)}$.

According to Theorem 3, $\min(\Gamma_1)\leqslant\beta u_Y^{\omega_2}(\mathbf{p},\mathbf{q})+\gamma\leqslant\max(\Gamma_1)$, where

$$\Gamma=\{\beta(R_2-R_1),\beta(T_2-T_1),\beta(S_2-S_1),\beta(P_2-P_1)\}.$$

Thus, $|\beta u_Y^{\omega_2}(\mathbf{p},\mathbf{q})+\gamma|\leqslant|\beta|\delta$, where $\delta=\max\{|R_2-R_1|,|S_2-S_1|,|T_2-T_1|,|P_2-P_1|\}$. Then $u_Y^{\omega_2}\leqslant\delta-\frac{\gamma}{\beta}$. $u_X^1=\frac{R_2(T_2-S_2)-(T_2-R_2)(\delta-\frac{\gamma}{\beta})}{(R_2-S_2)}$ is a solution of the following equations:

$$\begin{cases}u_Y=\delta-\dfrac{\gamma}{\beta},\\ (R_2-S_2)u_X+(T_2-R_2)u_Y=R_2(T_2-S_2).\end{cases}$$

Also, $u_X^2=\frac{R_2(T_2-S_2)-(R_2-S_2)(\delta-\frac{\gamma}{\beta})}{(T_2-R_2)}$ is the solution of the following equations:

$$\begin{cases}u_Y=\delta-\dfrac{\gamma}{\beta},\\ (T_2-R_2)u_X+(R_2-S_2)u_Y=R_2(T_2-S_2).\end{cases}$$

When $a_i^1\dfrac{\gamma}{\beta}>b_i^1, i\in\{1,2\}$, we have

$$[(R_1-S_1)(T_2-R_2)-(R_2-S_2)(T_1-R_1)]\frac{\gamma}{\beta}>(R_2-S_2)R_1(T_1-S_1)-(R_1-S_1)R_2(T_2-S_2)+\delta(R_1-S_1)(T_2-R_2),$$

$$[(R_1-S_1)(R_2-S_2)-(T_2-R_2)(T_1-R_1)]\frac{\gamma}{\beta}>(T_2-R_2)R_1(T_1-S_1)-(R_1-S_1)R_2(T_2-S_2)+\delta(R_1-S_1)(R_2-S_2).$$

Thus, $u_X^1>\bar{u}_X^{\omega_1}$, and $u_X^2>\bar{u}_X^{\omega_1}$. Since $u_X^1$ and $u_X^2$ is lower than player $X$'s utility when player $Y$ chooses ALLC strategy, the supremum of player $X$'s expected utility in $G_2$ is higher than it in $G_1$.

## 8 Proof of Corollary 5

For any $\phi, \chi > 1$, suppose player $X$ chooses extortion strategy $\mathbf{p} = \phi[(\mathbf{S}_X^{\omega_1} - P_1\mathbf{1}) - \chi(\mathbf{S}_Y^{\omega_1} - P_1\mathbf{1})] + \mathbf{p}_0$ in $G_1$. When player $Y$ choose the ALLC strategy, player $X$ get the supremum of its expected utility, $\bar{u}_X^{\omega_1}$, in $G_1$. $\bar{u}_X^{\omega_1}$ is the solution of the following equations:

$$\begin{cases} u_X - P_1 = \chi(u_Y - P_1), \\ (R_1 - S_1)u_X + (T_1 - R_1)u_Y = R_1(T_1 - S_1). \end{cases}$$

Thus, $u_X = \frac{\chi[R_1(T_1-S_1)-P_1(T_1-R_1)]+P_1(T_1-R_1)}{\chi(R_1-S_1)+(T_1-R_1)}$.

According to Theorem 3, $\min(\Gamma_2) \leqslant \phi[(u_X - P_2) - \chi(u_Y - P_2)] \leqslant \max(\Gamma_2)$, where

$$\Gamma_2 = \{\phi(1-\chi)(R_2 - R_1), \phi(S_2 - S_1) - \phi\chi(T_2 - T_1), \phi(T_2 - T_1) - \phi\chi(S_2 - S_1), \phi(1-\chi)(P_2 - P_1)\}.$$

Thus,

$$|(u_X - P_2) - \chi(u_Y - P_2)| \leqslant \max\{(\chi-1)|R_2 - R_1|, |S_2 - S_2| + \chi|T_2 - T_1|, |T_2 - T_1| + \chi|S_2 - S_2|, (\chi-1)|P_2 - P_1|\} \leqslant (\chi+1)\delta.$$

Then $u_X - P_2 - \chi(u_Y - P_2) \geqslant -(\chi+1)\delta$. $u_X^1 = \frac{\chi[R_2(T_2-S_2)-P_2(T_2-R_2)]+P_2(T_2-R_2)-(\chi+1)\delta(T_2-R_2)}{\chi(R_2-S_2)+(T_2-R_2)}$ is the solution of the following equations:

$$\begin{cases} u_X - P_2 - \chi(u_Y - P_2) = -(\chi+1)\delta, \\ (R_2 - S_2)u_X + (T_2 - R_2)u_Y = R_2(T_2 - S_2). \end{cases}$$

Also, $u_X^1 = \frac{\chi[R_2(T_2-S_2)-P_2(R_2-S_2)]+P_2(R_2-S_2)-(\chi+1)\delta(R_2-S_2)}{\chi(T_2-R_2)+(R_2-S_2)}$ is the solution of the following equations:

$$\begin{cases} u_X - P_2 - \chi(u_Y - P_2) = -(\chi+1)\delta, \\ (T_2 - R_2)u_X + (R_2 - S_2)u_Y = R_2(T_2 - S_2). \end{cases}$$

When $a_i^2\chi^2 + b_i^2\chi + c_i^2 < 0, i \in \{1,2\}$, we have $u_X^1 > \bar{u}_X^{\omega_1}$, and $u_X^2 > \bar{u}_X^{\omega_1}$. Since $u_X^1$ and $u_X^2$ is lower than player $X$'s utility when player $Y$ chooses the ALLC strategy, the supremum of player $X$'s expected utility in $G_2$ is higher than it in $G_1$.

## 9 Proof of Corollary 6

When player $Y$ chooses the always defect (ALLD) strategy, $(u_X^{\omega_i}, u_Y^{\omega_i})$ is on $(T_i - P_i)u_X^{\omega_i} + (P_i - S_i)u_Y^{\omega_i} = P_i(T_i - S_i)$ in $G_i, i \in \{1,2\}$. For any $\phi, \chi > 1$, suppose player $X$ chooses generous strategy $\mathbf{p} = \phi[(\mathbf{S}_X^{\omega_1} - R_1\mathbf{1}) - \chi(\mathbf{S}_Y^{\omega_1} - R_1\mathbf{1})] + \mathbf{p}_0$ in $G_1$. When player $Y$ choose the ALLD strategy, player $X$ get the infimum of its expected utility, $\underline{u}_X^{\omega_1}$, in $G_1$. $\underline{u}_X^{\omega_1}$ is the solution of the following equations:

$$\begin{cases} u_X - R_1 = \chi(u_Y - R_1), \\ (T_1 - P_1)u_X + (P_1 - S_1)u_Y = P_1(T_1 - S_1). \end{cases}$$

Then $u_X = \frac{\chi[P_1(T_1-S_1)-R_1(P_1-S_1)]+R_1(P_1-S_1)}{\chi(T_1-P_1)+(P_1-S_1)}$.

According to Theorem 3, $\min(\Gamma_2) \leqslant \phi[(u_X - R_2) - \chi(u_Y - R_2)] \leqslant \max(\Gamma_2)$, where

$$\Gamma_2 = \{\phi(1-\chi)(R_2 - R_1), \phi(S_2 - S_1) - \phi\chi(T_2 - T_1), \phi(T_2 - T_1) - \phi\chi(S_2 - S_1), \phi(1-\chi)(P_2 - P_1)\}.$$

Thus,

$$|(u_X - R_2) - \chi(u_Y - R_2)| \leqslant \max\{(\chi-1)|R_2 - R_1|, |S_2 - S_2| + \chi|T_2 - T_1|, |T_2 - T_1| + \chi|S_2 - S_2|, (\chi-1)|P_2 - P_1|\} \leqslant (\chi+1)\delta.$$

Then we have

$$u_X - R_2 - \chi(u_Y - R_2) \geqslant -(\chi+1)\delta.$$

Let $u_X^1 = \frac{\chi[P_2(T_2-S_2)-R_2(P_2-S_2)]+R_2(P_2-S_2)-(\chi+1)\delta(P_2-S_2)}{\chi(T_2-P_2)+(P_2-S_2)}$ be the solution of the following equations:

$$\begin{cases} u_X - R_2 - \chi(u_Y - R_2) = -(\chi+1)\delta, \\ (T_2 - P_2)u_X + (P_2 - S_2)u_Y = P_2(T_2 - S_2). \end{cases}$$

Also, let $u_X^2 = \frac{\chi[P_2(T_2-S_2)-R_2(T_2-P_2)]+R_2(T_2-P_2)-(\chi+1)\delta(T_2-P_2)}{\chi(P_2-S_2)+(T_2-P_2)}$ be the solution of the following equations:

$$\begin{cases} u_X - R_2 - \chi(u_Y - R_2) = -(\chi+1)\delta, \\ (P_2 - S_2)u_X + (T_2 - P_2)u_Y = P_2(T_2 - S_2). \end{cases}$$

When $a_i^3\chi^2 + b_i^3\chi + c_i^3 < 0, i \in \{1,2\}$, we have $u_X^1 > \underline{u}_X^{\omega_1}$, and $u_X^2 > \underline{u}_X^{\omega_1}$. Since $u_X^1$ and $u_X^2$ is lower than player $X$'s utility when player $Y$ chooses the ALLD strategy, the infimum of player $X$'s expected utility in $G_2$ is higher than it in $G_1$.

## References


**1.** Press, W. H. & Dyson, F. J. Iterated prisoner's dilemma contains strategies that dominate any evolutionary opponent. *Proc. Natl. Acad. Sci.* **109**, 10409–10413 (2012).
